# Autonomous swab robot for naso- and oropharyngeal COVID-19 screening

**DOI:** 10.1038/s41598-023-50291-1

**Published:** 2024-01-02

**Authors:** Simon Haddadin, Dirk Wilhelm, Daniel Wahrmann, Fabio Tenebruso, Hamid Sadeghian, Abdeldjallil Naceri, Sami Haddadin

**Affiliations:** 1 Chair of Robotics and Systems Intelligence, School of Computation, Information and Technologies, Munich Institute of Robotics and Machine Intelligence, Technical University Munich, Munich, Germany; 2 School for Medicine and Health, Klinikum rechts der Isar, Department of Surgery, Technical University Munich, Munich, Germany; 3 Franka Emika GmbH, Munich, Germany

**Keywords:** Population screening, Biomedical engineering

## Abstract

The COVID-19 outbreak has triggered a global health and economic crisis, necessitating widespread testing to control viral spread amidst rising cases and fatalities. The recommended testing method, a combined naso- and oropharyngeal swab, poses risks and demands limited protective gear. In response to the COVID-19 pandemic, we developed and tested the first autonomous swab robot station for Naso- and Oropharyngeal Coronavirus Screening (SR-NOCS). A force-sensitive robot running under a Cartesian impedance controller is employed to drive the swab to the sampling area. This groundbreaking device underwent two clinical studies-one conducted during the initial pandemic lockdown in Europe (early 2021) and the other, more recently, in a public place after the pandemic had subsided earlier in the year 2023. In total, 52 patients suspected of COVID-19 infection were included in these clinical studies. The results revealed a complete positive correlation between autonomous and manual sampling. The test subjects exhibited a high acceptance rate, all expressing a willingness to undergo future tests with SR-NOCS. Based on our findings, such systems could enhance testing capabilities, potentially conducting up to 300 tests per robot per day with consistent precision. The tests can be carried out with minimal supervision, reducing infection risks and effectively safeguarding patients and healthcare workers.

## Introduction

The outbreak of COVID-19 has led to an unprecedented health and economic crisis^1^. In more than 200 countries, areas, or territories over 700 million confirmed cases have been registered with almost 7 million fatalities^2,3^. Widespread testing is currently the most essential measure to control viral spread^4–6^. Combined nasopharyngeal (NP) and oropharyngeal (OP) swab is the recommended choice of sampling, although it constitutes a high-risk procedure and requires personal protective equipment which subject to shortage^7–9^. As we breathe, we take our respiratory system and health for granted. However, the lung is a vital organ vulnerable to airborne infection and injury. Respiratory diseases are the leading causes of death and disability in the world, taking away the lives of over 3 million people annually^10^. While chronic diseases and long-term lung injury are underlying reasons for most breathing disabilities, severe acute respiratory syndrome (SARS) is induced by infection with the Coronavirus and was first described in 2002^11^. Coronavirus SARS-CoV-2, responsible for the outbreak of COVID-19, has brought the world to an unprecedented health and economic crisis. It emerged in Wuhan, China at the end of 2019 and claimed more than 3000 deaths in Hubei, China within a few weeks^1^. SARS will go into the medical records as the first new pandemic disease to sweep through the world population in the 21st century^12^. As we enter the third year since the start of COVID-19, deep concerns are being expressed by the World Health Organization (WHO) about the rapid escalation and global spread of infection^13^. In the first weeks of the COVID-19 outbreak in Germany (Europe), we witnessed a near-exponential growth in the number of new cases reaching almost every country and territory worldwide. At that time, more than 200 countries were affected and between 2,319,0662 and 2,478,6343 cases were identified. The estimated number of associated deaths ranges between 157,9702 and 170,3893.

**Figure 1:**
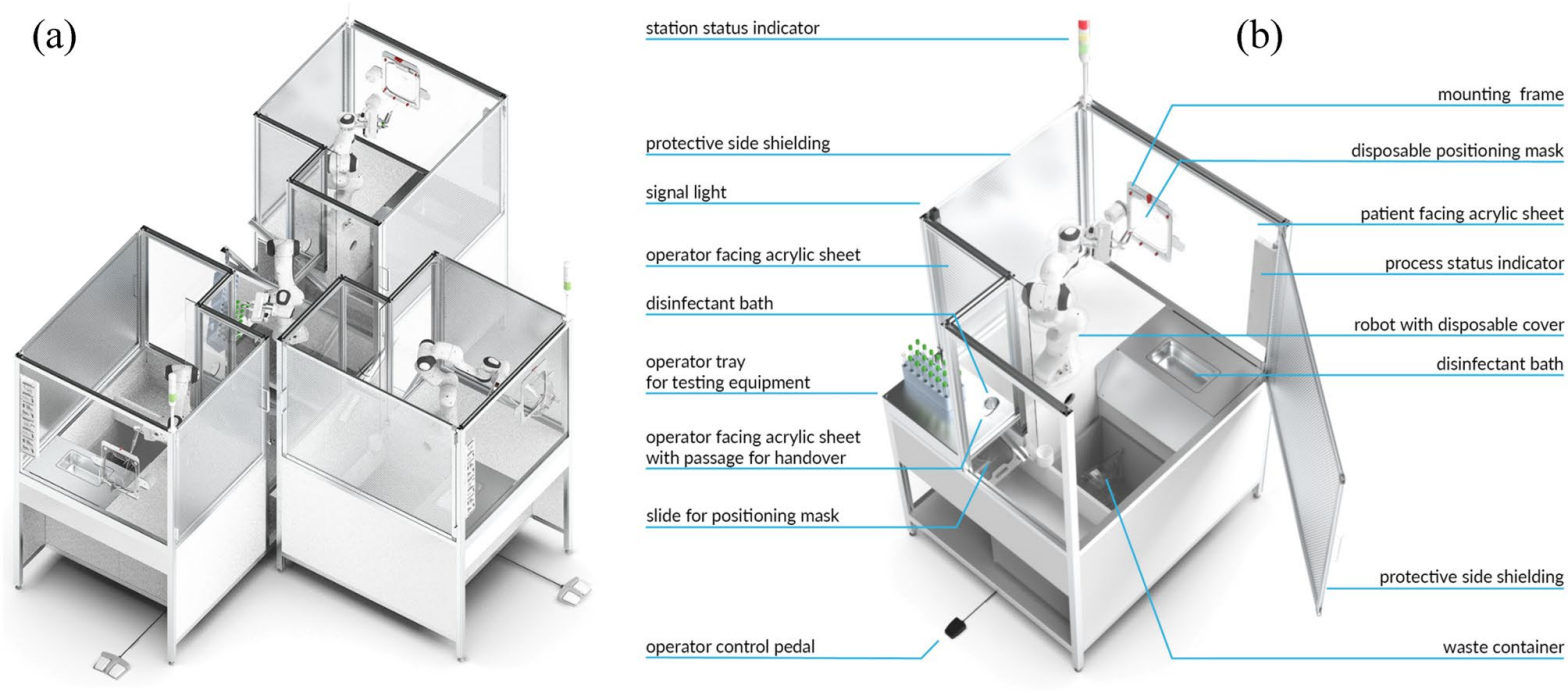
Swab robot for NP and OP Covid-19 screening. (**a**) Modular swab stations that can be easily interconnected and installed in a compact area. (**b**) Detailed specifications of the swab station (see also “Methods” section).

### COVID-19 and the healthcare workforce

Multiple coronavirus outbreaks lie already behind us^14,15^. There are currently many recommendations and different types of vaccines (Messenger RNA (mRNA) vaccine, Vector vaccine, Protein subunit vaccine), but these do not prevent the virus from spreading between individuals without side effects. According to the experience gained by the 2002 SARS outbreak^14^, emphasis should be placed on protecting healthcare workers (HCWs), as they face an elevated risk of exposure to infectious diseases^16^. In the first weeks of the COVID-19 outbreak in Wuhan approximately 1725 front-line HCWs were infected by SARS-CoV-215^17^, while 2026 infections among HCWs were counted in Italy by the beginning of April 2020^18^. In response, an early study recommended prevention and control actions^19^ and pointed out that ensuring the safety of HCWs not only ensures patient care but also prevents further transmission of the virus to the general population^16,17^. Among the highest risk factors for infection are (i) working at high-risk departments, (ii) longer exposure time, and (iii) suboptimal hand hygiene after contacting patients^20^, revealing the risks HCWs are willing to take^12^. Increasing awareness of personal protective equipment (PPE) and response would be essential in lowering the risk of infection for HCWs^21,22^. Non-symptomatic may spread the virus possibly even by aerosol and direct fomite transmission. Most frequent infections come from human errors in adhering to hygiene procedures^17,21,23–26^. The primary goal of public health measures is to prevent person-to-person spread of disease by isolation and quarantine, social distancing, and community containment^27^ and the proper use of PPE. Knowledge of incidence and prevalence rates of COVID-19, as well as certainty on who is infected, are critical to an effective response to the disease and require large-scale diagnostic testing^28^.

### The importance of COVID monitoring

Testing, testing, testing has been the mantra repeated by Director-General Tedros Adhanom Ghebreyesus^4,29^. Widespread diagnostic testing, isolation of the infected, contact tracing, and consequent quarantining of those contacts seem to have been key in South Korea’s and Singapore’s work to suppress virus spread^4–6^. However, testing capacities worldwide show significant differences: the United Arab Emirates performed more than 12,738 tests per million (p.m.) inhabitants and South Korea more than 6148 p.m. while the United States of America conducted less than 314 tests p.m. For initial diagnostic testing for SARS-CoV-2, CDC recommends collecting and testing oro- and nasopharynx specimens. The NP specimen is the preferred choice for swab-based SARS-CoV-2 testing and is now recommended by the CDC as the only assessment site. If both swabs are used, NP and OP specimens should be combined at collection into a single vial^4,30^. Collecting an NP specimen requires an HCW to put on personal protective equipment (PPE) and insert a swab deep into the nasal cavity and throat near the patient, risking infection. While conducting NP and OP swabs HCWs cannot respect all recommendations as social distancing^31,32^. As such, the process consumes PPE and time, two resources that are in short supply for providers contending with the COVID-19 pandemic^7–9^. Meanwhile, rapid expansion of testing has taken place, but there are significant hurdles for testing in ambulatory clinics, emergency departments, and hospitals. Testing capacities pose risks due to overcrowding and nosocomial transmission. Meanwhile, testing capacities worldwide are already at their limit^4–6,8,9,33–36^. One obvious countermeasure is self-testing with nasal swabs which has indeed been shown to be a viable screening option in cases of influenza but is estimated non-reliable in terms of diagnostic efficiency^37–39^.

### Swab robots approach for screening after the COVID-19 pandemic

Automating swab collection and ensuring physical separation between healthcare workers (HCWs) and potentially infected patients presents a viable solution for supporting HCWs. An automated process would not only ensure consistent adherence to hygienic measures^33,40^, but also facilitate a faster screening process, enabling larger-scale surveys. Consequently, we propose that innovative robot systems, characterized by their tactile sensitivity and compliance, have the potential to perform swab procedures with comparable efficiency and precision to HCWs while being accepted by test subjects as a viable alternative. In this section, we explore the latest research on swab robotic systems, discussing their advancements and capabilities in sample collection. In response to the recent pandemic, numerous papers have emerged that tackle the development of robotic systems aimed at enhancing sample collection for COVID-19 testing. For example, researchers introduced quickly deployable robotic systems with enhanced dexterity in sampling, resulting in improved efficiency and reduced workload for healthcare professionals^41,42^. Another approach focused on remote systems as a safer measure^43,44^ introducing remotely controlled OP and NP swab sampling robots designed explicitly for COVID-19 prevention. These robots enable safer and more efficient sample collection, effectively reducing the risk of virus transmission. Another notable focus in robotic sample collection for COVID-19 testing involved the visual servo control of a NP swab sampling robot^45^. Visual feedback enables precise navigation and sampling, thereby enhancing the accuracy and reliability of the testing process. Additionally, a novel approach was introduced that enhances robotic manipulations by incorporating a spherical wrist with hybrid motion-impedance control^46^. This mechanical device gives the robot three degrees of freedom, enabling it to perform complex manipulation tasks. However, it is essential to note that the mentioned work lacks experimental validation and real-world implementation and validation. Therefore, further assessment is required to evaluate the accuracy, robustness, and performance of the control strategy in practical applications. Regarding ergonomics, researchers introduced a prototype of a pneumatic-actuated soft nasal swab (V-tube) for COVID-19 screening^47^. The authors claim that the soft swab design enhances patient comfort during sample collection. Additionally, there has been a focus on the design and control of a highly redundant rigid-flexible coupling robot for OP swab sampling in COVID-19 cases^48^. This robot assists medical professionals in sample collection, improving efficiency and reducing the risk of infection. Moreover, autonomous approaches have been introduced for the OP-Swab Robot System^49,50^. These systems automate the swabbing process, minimizing human contact and reducing the risk of virus transmission while enhancing the efficiency and accuracy of sample collection. Regarding safety, a force restriction mechanism has been implemented in a NP swab sampling robot^51^. This mechanism ensures that excessive force is prevented during sample collection, thereby ensuring patient safety. Overall, these works demonstrate notable advancements in robotic sampling for COVID-19 testing. However, certain limitations are left. These include the need for calibration, accounting for variability in anatomical structures, training requirements, design complexity, scalability, and the necessity for additional experimental validation and real-world implementation (additional information regarding the related works is available in Supplementary Table S1). In the present work, we address many of these limitations, and by recognizing the importance of overcoming them foster the development of more efficient, reliable, and user-friendly robotic systems for COVID-19 sample collection.

### Our proposed swab robot for screening

**Figure 2:**
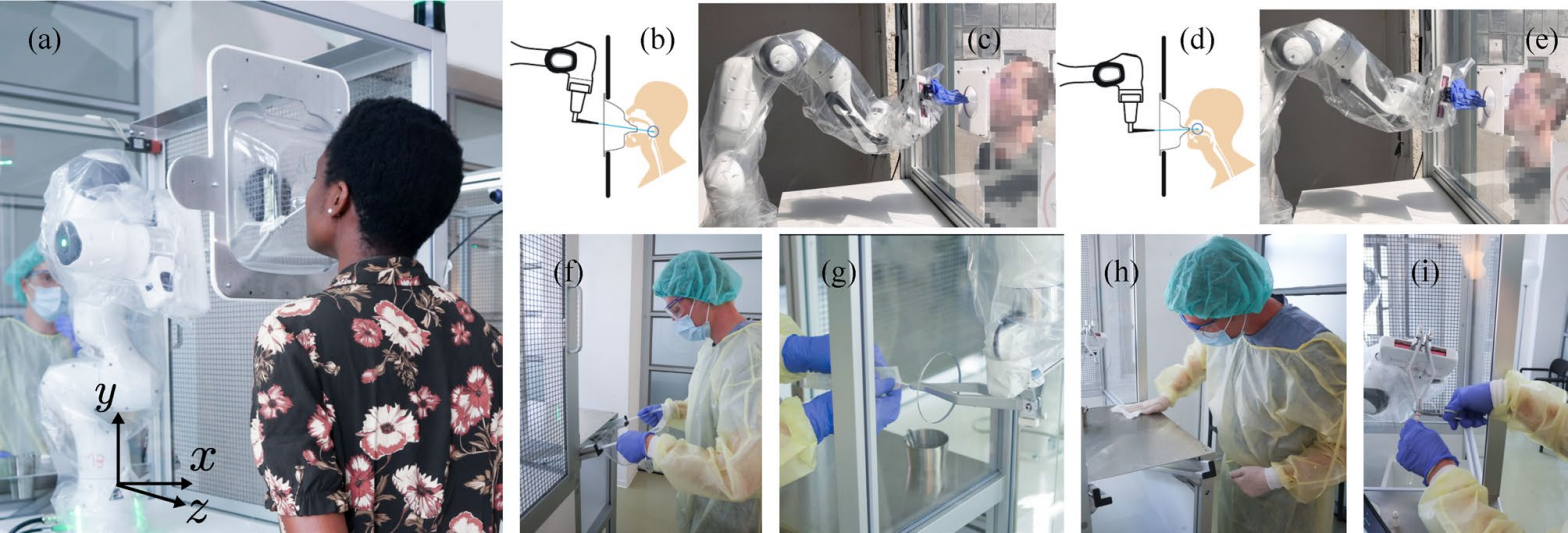
The swab robot for NP and OP COVID-19 screening and example procedure. Photos were taken during the actual study and in a pre-test. (**a**) Subject during NP swabbing. (**b**,**c**) A depiction and actual robotic system for the OP swab procedure, respectively. (**d**,**e**) A depiction and actual robotic system for the NP swab procedure, respectively. (**f**–**i**) Snapshots of a healthcare worker supervising the station and performing the necessary support actions, such as handling new material, disinfecting, and sorting samples.

**Figure 3:**
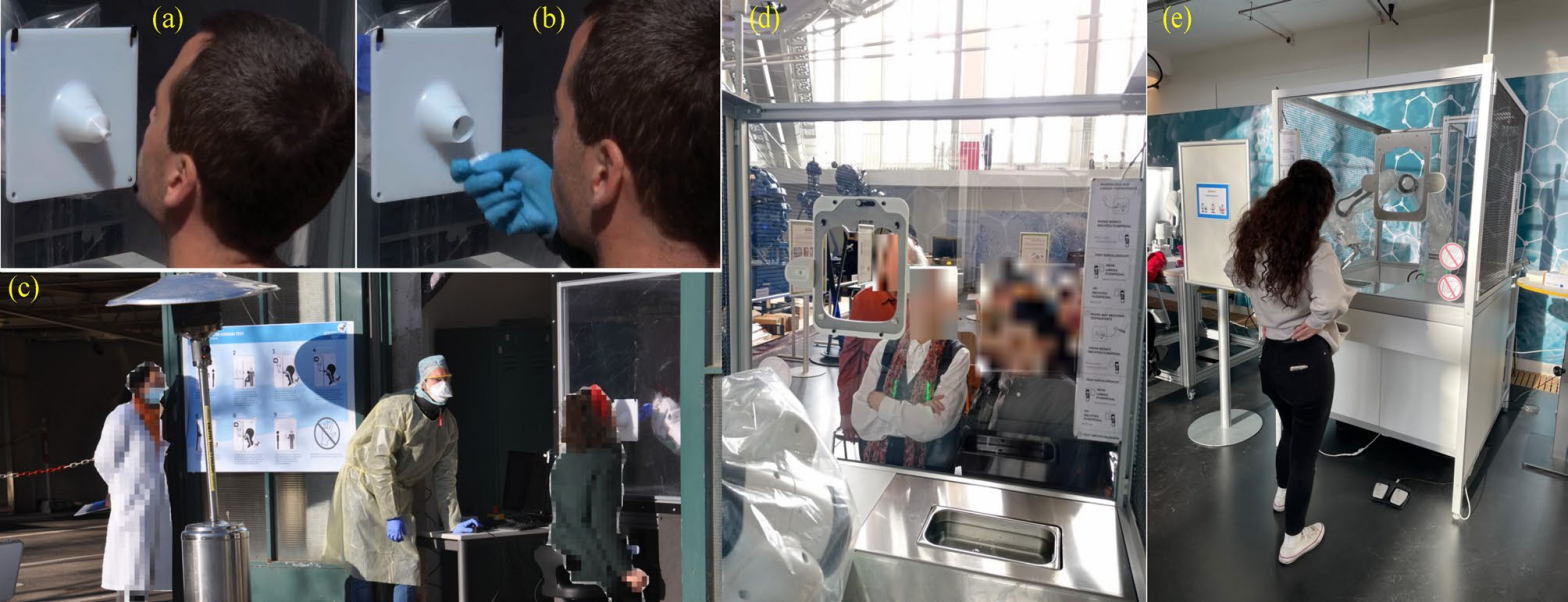
Clinical studies within Experiment 1 and 2. (**a**,**b**) The nose and throat disposable fixation used during swab sampling in the clinical study. The fixture design can be adapted for both NP (**a**) and OP (**b**) screening procedures. (**c**) Clinical study within Experiment 1. The autonomous swab robot for NP / OP Corona screening is being performed for one of the test subjects (right in **c**), while the operator (center of c, in fully protective equipment) observes with his finger ready on the control device (see “Test workflow” method); a hospital physician (left in **c**) observes the test from a safe distance. (**d**,**e**) Public visitors engaging with the swabbing robot station at the *Deutsches Museum* in Munich within Experiment 2. (**d**) depicts an internal view, while (**e**) shows an external perspective. The image is partially blurred to preserve anonymity.

For the above reasons, the first autonomous swab robot for NP and OP coronavirus screening (SR-NOCS) was developed (see Figs. 1 and 2). It was designed with a focus on portability, high-throughput ability, and with less contact between patient and HCW, to be deployable at scale within a short time frame. The idea is to perform the swab procedure autonomously by a robot strictly following the given contact restrictions and hygienic rules to protect HCWs, effectively. A plexiglass wall isolates the robot from the patient, who sits on a chair (or standing) and controls the procedure via a pedal near the patient’s feet. Before the swab is collected, the patient has to place his/her nose or mouth on a disposable fixation (Fig. 3a,b) which is changed between tests for hygienic safety reasons - and indicates conformity by pressing the pedal (see Figs. 1 and 2).

One of the primary obstacles in implementing autonomous screening with NP and OP swabs lies in the necessity for a motion system that is both highly tactile sensitive and adaptable, tailored to the unique anatomy of each individual undergoing testing. Conventionally, tasks demanding precision and repetitive actions often deploy position-controlled robots renowned for their inherent rigidity. Nevertheless, in this specific swab sampling experiment, we employed the compliant Franka Emika robot arm^52^ to introduce flexibility and adaptability into the system. This robot is equipped with joint torque sensors in each joint, enabling precise control over its compliance, contact sensitivity, and contact forces with its environment. This allows the robot to adapt its motion to each subject based on the sensed interaction forces and its compliance, resulting in a human-like swab procedure (see “Methods” for more details). For this prototype, the focus was set on the autonomous collection of NP and OP samples. A prospective observational clinical study was carried out with the primary objective of examining differences in detecting SARS-CoV2 from specimen sampling either by manual or by autonomous NP and OP swab collection (see Figs. 2f–i and 3c). To isolate the swabbing procedure in this comparison, all other processes (e.g. station disinfection, loading, and unloading of the cotton bud swab) were performed with human assistance but could be further automated.

## Results

Two significant experiments were undertaken in this study. The first experiment, took place on April 1 and 2, 2020, involving healthcare workers (HCWs; Fig. 3c), as elaborated below. The second experiment was conducted at the Deutsches Museum in Munich from January 16, 2023, to April 10, 2023 (Fig. 3d,e), with randomly selected visitors. Below, we provide a detailed account of the outcomes of both experiments.

### Experiment 1

Enrollment occurred on April 1st and 2nd 2020 in Munich’s central population. 31 HCWs of the university clinic Klinikum rechts der Isar of the Technical University Munich were tested - demographic and clinical variables are listed in Table 1. All included test subjects were indicated for testing irrespective of the presented study, either due to symptoms suspicious for COVID-19 (such as fever, dry cough, dyspnea, headaches, joint pain, sneezing) and/or post-exposure surveillance. As test subjects received both tests one after the other, no randomization was necessary. Two swabbing tests were performed for each candidate, one manually and one by the robot to verify the consistency of the results. All swabs were tested by real-time reverse transcriptase polymerase chain reaction (RT-PCR) for SARS-CoV2^6^. Paired specimens were processed parallel to the clinical laboratory as part of routine patient specimen testing. Paired samples were collected from 31 adults; 1 (3.2 %) individual tested positive for Coronavirus by rRT-PCR, whereas 30 (96.8 %) patients were free of SARS-CoV-2 during sampling. Statistical analysis revealed a complete positive correlation of 1.0 when analyzed using a double-sided Spearman test (further details are illustrated in Table 2).

**Table 1:** Demographic data of both experiments.

Characteristic	Value
(a) Demographic data of the 31 test subjects in a clinical study of Experiment 1	
Average age (range) [years]	37.2 (22–62)
Sex ratio male:female (percentage)	12:19 (38.7:61.3)
Number of HCWs among test subjects (percentage)	24 (83.9)
Number of physicians among test subjects (percentage)	7 (22.6)
(b) Demographic data of the 21 test subjects in clinical study of Experiment 2	
Average age (range) [years]	33.6 (19–75)
Sex ratio male:female (percentage)	13:8 (61.9:38.1)

**Table 2:** Comparison between the manual test performed by HCWs and the SR-NOCS.

Count		Robotic test 1 = negative, 2 = positive		Total	
		1	2		
Standard test 1 = negative; 2 = positive	1	30	0	30	
	2	0	1	1	
Total		30	1	31	

Symmetric measures	Value	Asymptotic standardized error$^a$	Approximate $T^b$	Approximate significance	Total
Ordinal by ordinal	Kendall’s tau-b	1.000	0.000	1.052	293
	Spearman correlation	1.000	0.000c		
Interval by interval	Pearson’s R	1.000	0.000c		
N of valid cases		31			

Correlations			Standard test 1 = negative; 2 = positive	Robotic test 1 = negative, 2 = positive	
Spearman’s rho	Standard test	Correlation coefficient	1.000	1.000**	
	1 = negative	Sig. (2-tailed)			
	2 = positive	N	31	31	
	Robotic test	Correlation coefficient	1.000**	1.000	
	1 = negative	Sig. (2-tailed)			
	2 = positive	N	31	31	

The statistical analysis applying a 2-sided Spearman correlation revealed a completely positive correlation.

$^{\text {a}}$Not assuming the null hypothesis.

$^{\text {b}}$Using the asymptotic standard error assuming the null hypothesis.

$^{\text {c}}$Based on normal approximation.

**Correlation is significant at the 0.01 level (2-tailed).

**Figure 4:**
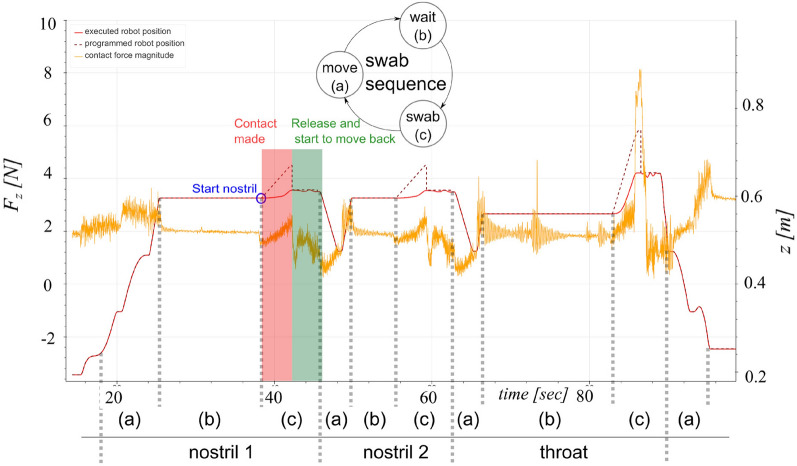
Robot force and position plot over time during a test procedure. A complete test routine (test subject 26) is shown including all three swab sequences and a handover motion at the end of the procedure. In each sequence, the robot moves to the swab’s initial position (**a**), waits for confirmation from the patient and operator (**b**), and performs the swab motion (**c**). The programmed and executed robot positions indicate horizontal displacements in the robot’s forward direction in world coordinates (towards the test subject). The divergence between them indicates a contact and coincides with an increased value of the estimated contact force.

**Figure 5:**
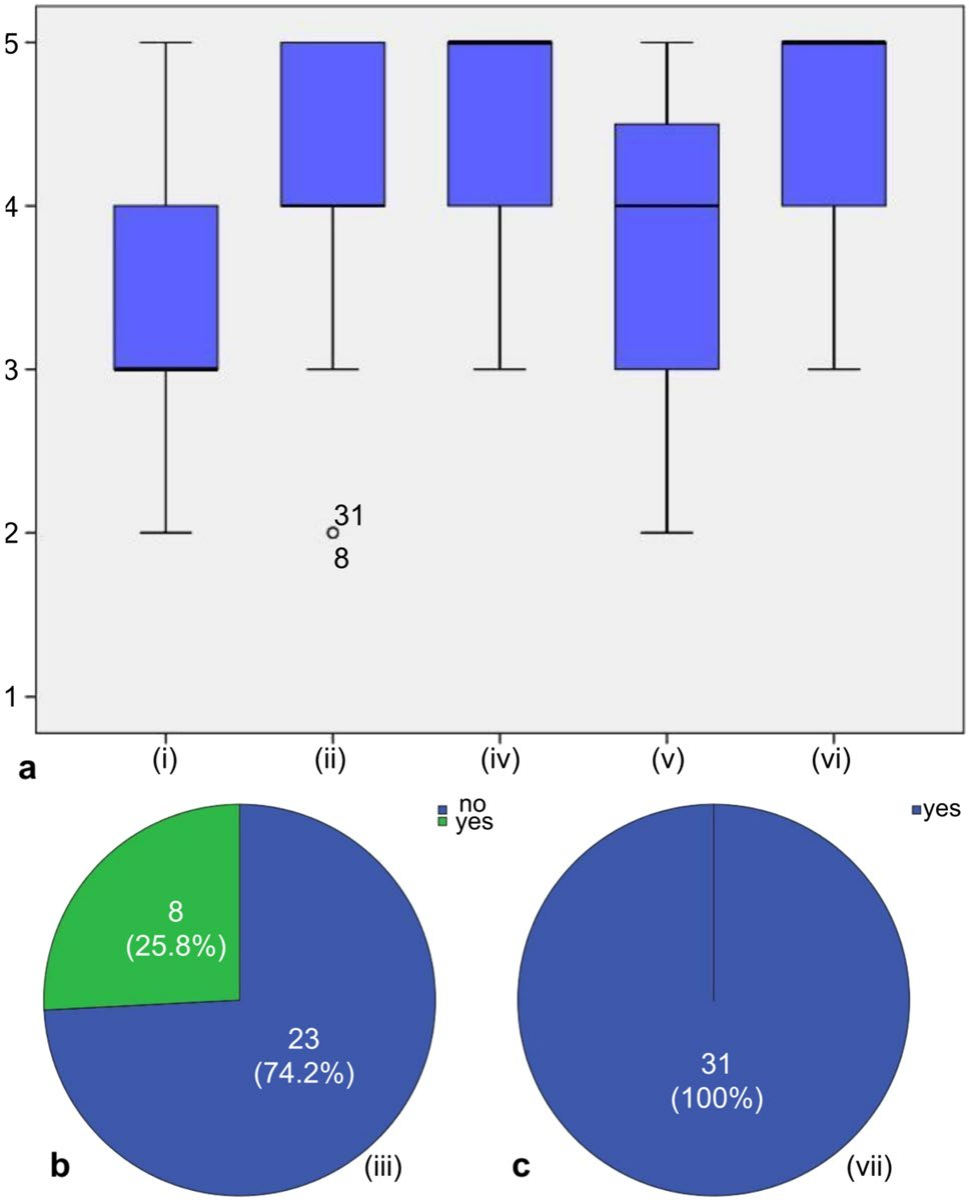
Test subjects’ survey. (**a**) A box plot representation of the answers to the Likert scale questions, from 1 (least favorable assessment) to 5 (most favorable assessment). Thick (center) lines show the medians; box limits indicate the correlated variances; whiskers visualize the minimum and maximum ratings (n = 31). (**b**,**c**) The number of yes/no answers for questions (iii) and (vii), respectively. The respective pieces display percentages of the whole.

A typical test workflow (see “Methods”) took 5–6 min, including 1–2 min for introduction and 2 min for the test subject survey; the disinfection procedures and change of fixtures and gloves took 2–4 min (processes may have taken place simultaneously). The SR-NOCS’s test conduction time was measured from the operator’s initiation of the test procedure until the handover of the swab probe. It was 108 s on average, whereas the shortest test took 60 s and the longest 242 s. Figure 4 illustrates the force and position profiles of the robot over time during a single test procedure for a representative subject. Tests that took longer than 3 min (4 out of 31) included a repetition of at least one swab initiated by the operator or the test subject. It is worth mentioning that the last half of the tests were performed without any repetition. This might be attributed to the lack of experience of the hospital employees with the system at the beginning, which improved over time. Another factor in the variance of test conduction times was the extensive interactions between test subjects and hospital employees during tests (see Fig. 4, Table 3). Besides that, a test subject survey was conducted. The questions included five Likert scale questions, from 1 (the least favorable assessment) to 5 (the most favorable assessment), and two yes/no questions; which are reported in Table 4. As revealed by the survey, 7 out of 31 test subjects (22.6 %) graded the autonomous swab more uncomfortable. In contrast, the majority of patients found it either equal (13/31, 41.9 %), rather comfortable (7/31, 22.6 %), or even more comfortable (4/31, 12.9 %) than the manual procedure. For the same question, no differences were observed when comparing female and male patients (p = 0.205), nor when differentiating between physicians and other HCWs (p = 0.595). Most test subjects perceived a high level of safety with a median value of 4 (variance 0.806) and 2 out of 31 patients highlighted safety concerns (value 2). 74.2 % (23/31) of test subjects did not indicate any general concern during the autonomous test, while 8 subjects, mostly female non-physicians, indicated some kind of reservations. All test subjects regarded the autonomous swab robot (median value 5, variance 0.318) and robotic devices (median value 5, variance 0.303) as very helpful. No participant assessed robotic support functionalities as less or not helpful/essential (value 1 or 2) and only one participant responded neutrally (value 3) to this topic. All test subjects (31/31) would repeat a test by SR-NOCS in the future. The majority of (27/31) test subjects considered testing a relevant risk for HCWs (median value 4, variance 1.013), while 4 out of 31 test subjects estimated the risk to be low (value 2 on the Likert scale). More details are illustrated in Fig. 5.

**Table 3:** Performance of the SR-NOCS screening procedure.

Variable	Value	Comment
Programmed test duration [s]	41.9	Duration of an ideal test excluding waiting times. It is composed of robot motions as well as swabs and corresponds to all periods (a) and (c) in Fig. 5
Test conduction time (mean | SD | min | max) [s]	108 | 39 | 60 | 242	Recorded by the robot from the moment the robot motion started the test until it was ready for sample handover, including interruptions and restarts due to explanations, clarifications, errors or repetitions, for all 31 tests
The number of tests with repeated steps	4	When a swabbing procedure (left/right nostril or mouth) had to be restarted once or several times due to either an incorrect start by the operator or an unexpected reaction by the test subject (e.g. moving away from the station). Corresponds to test subjects n$^\circ$1 (3 swab repetitions), n$^\circ$4 (2 swab repetitions), n$^\circ$13 (2 swab repetitions), and n$^\circ$16 (1 swab repetition)
Test conduction time for tests without repetitions (mean | SD | min | max) [s]	99 | 28 | 60 | 177	Includes waiting periods and repetitions, for all 27 tests without repeated swabs
Test conduction time for tests with repetitions (mean | SD | min | max) [s]	171 | 55 | 109 | 242	Includes waiting periods and repetitions, for all 4 tests with repeated swabs
Shortest time between tests [min]	4	The minimum time between two consecutive test starts (minute accuracy) for all 31 tests. It includes all interactions with the test subject (see “Methods”). A 4-min difference in test starts took place once between test n$^\circ$4 and test n$^\circ$5 while there were 8 occurences of 5-min differences between test starts
Maximum relative contact forces* (mean | SD | min | max) [N]	5.1 | 1.3 | 2.6 | 7.2	Maximum magnitude of the relative force* along a whole test, for all 31 tests

See “Methods”.

**Table 4:** Test subject survey questions of Experiment 1.

Question	Answer options
(i) How did you find the automated swab compared to the conventional swab?	Likert scale from 1 (less comfortable) to 5 (more comfortable)
(ii) Did you feel safe during the test?	Likert scale from 1 (unsafe) to 5 (very safe)
(iii) Did you have any concerns during the test?	Yes/no
(iv) Do you think such automated tests with a robot system are essential/helpful measures to contain the corona crisis?	Likert scale from 1 (not helpful at all) to 5 (very helpful)
(v) How do you estimate the risk of infection for medical professionals with conventional test procedures?	Likert scale from 1 (very low) to 5 (very high)
(vi) Do you think robotic systems could be helpful elsewhere in the context of the Corona crisis?	Likert scale from 1 (surely not) to 5 (surely yes)
(vii) Would you use this test procedure again in the future?	Yes/no

### Experiment 2

**Figure 6:**
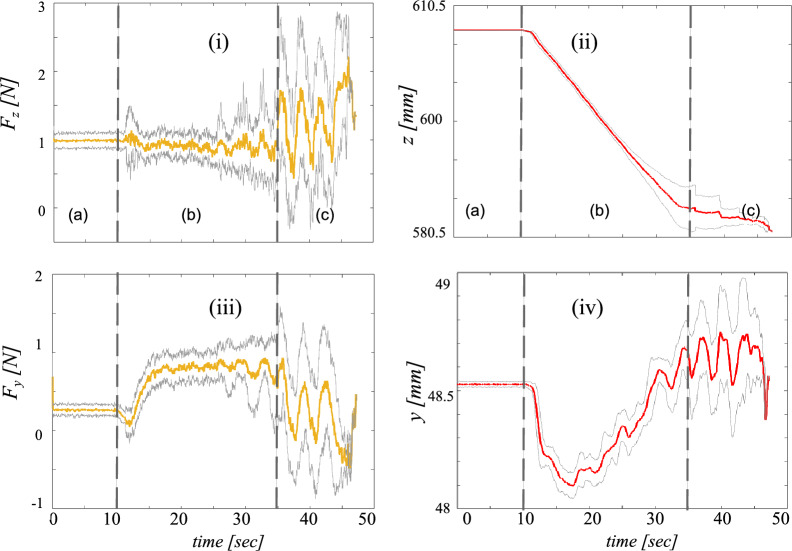
Robot force and position data within subject Coronal plane (y- and z-axes) during OP swab procedure conducted within experiment 2. Orange and red bold lines represent the mean and gray lines represent the standard deviation across 21 subjects. (i and iii) panels represent force data and (ii and iv) panel represents position data.

As part of the second experiment, we conducted a study at a public location, specifically the *Deutsches Museum*, Munich, the world’s largest science and technology museum (Fig. 3d,e). Twenty-one subjects participated in this phase, and the protocol and workflow of the previous experiment were followed. In this study, only the OP swab procedure was performed. Subjects were asked to do the test manually after the experiment and report the results when positive. In total, 21 paired robot and manual swabbing samples were also processed in parallel in the clinical laboratory as part of routine patient specimen testing. No individual tested positive for Coronavirus by rRT-PCR, indicating that all (21, 100 %) patients were free of SARS-CoV-2 during sampling in this experiment. The experimental testing procedure (as outlined in the “Methods”, similar to Experiment 1) required slightly less time than Experiment 1, as only the implementation of the OP procedure has been conducted. On average, this process took 37 s, with the quickest test completed in 16 s and the longest lasting 47 s. Figure 6 depicts the mean and standard deviations of force and position profiles displayed by the robot during the test procedures for all subjects. Upon initial observation, our objective was to assess the repetitiveness of the motion (position) and physical interaction (forces) data profiles acquired during the swabbing procedure (Fig. 6). The position and force profiles are consistent across all subjects, with a maximum deviation in position and force of about 5 mm and 1 N (100 g), respectively. Experiment 2 demonstrates the robot’s ability to precisely replicate even contact-rich swabbing procedures for different subjects consistently from the first to the last subject. In the post-questionnaire data collected from subjects, as well as input from the booth supervisor, our primary focus was to assess the extent to which subjects adhered strictly to the protocol. Additionally, we evaluated the level of assistance provided by the onboard operator and the clarity of the instructions given for the subjects to follow. Our findings revealed that most subjects (20/21, 95.24 %) comprehended the training material, while only one subject (1/21, 4.76 %) encountered difficulties. Furthermore, our results indicated that all subjects (21/21, 100 %) reported the necessity of operator presence to assist them during their tests. In this experiment, it is worth mentioning that none of the trials were repeated. This indicates that the participants and the booth operator adhered properly to the established swabbing station protocol. Certainly, our results revealed that most subjects adhered to the defined hygiene concept (15/21, 71.43 %), whereas a smaller portion (6/21, 28.57 %) did not fully comply with the hygiene protocol. While engaging with the swabbing booth, the majority of subjects (20/21, 95.23 %) promptly informed the operator in the event of touching the acrylic sheet. Additionally, all subjects adhered to the designated testing protocol of the booth.

**Figure 7:**
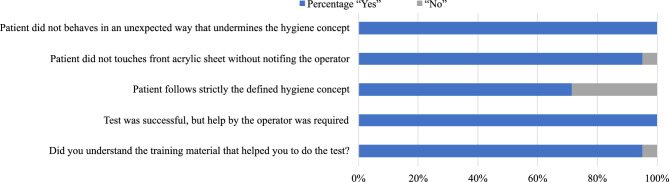
Post-experiment 2 questionnaire response rates, reflecting the extent to which participants followed the established protocol.

Overall, the response rates indicate that participants diligently followed the instructions and comprehended them effectively, aided by the assistance provided by the booth supervisor, as depicted in Fig. 7. The primary outcome of the second experiment is the validation of consistent repeatability of the robot’s motion and interaction (applied forces) behavior during the OP swab procedure. It is worth noting that in contrast to any other existing work, Experiment 2 was conducted in public. In summary, the results affirm the system’s feasibility and strict adherence to the prescribed protocol during interactions of humans with the swabbing robot.

## Discussion

In the current COVID-19 crisis, it again became evident how dependent society is - even in the digital era - on the physical presence of countless people at neuralgic intersections. This applies especially to the healthcare sector, which even before this crisis was at a critical level. COVID-19 has led to increasingly exhausted capacities within healthcare sectors in several countries. We believe this is an outdated approach, as protecting indispensable HCWs is paramount and should be supported by autonomous systems. This way, HCWs can focus on completing more decisive and human-inherent tasks. Even considering the availability of PPE and profound training in its usage, non-exposure is a measure second to nothing as it reliably reduces the risk of contamination to zero. An avoidance strategy should be chosen whenever possible and ethically justifiable. In this context supporting HCWs with an autonomous swab robot collecting samples could be a substantial measure. Two primary clinical studies were conducted: the first one during the early European lockdown in 2020, and the second one in early 2023 in a public setting after the pandemic had subsided. To the best of the authors’ knowledge, this work has been the first and only robot swabbing system that has been clinically tested during the first European lockdown. Moreover, a later version has been validated in a public setting at the world’s largest science and technology museum in Munich. During the recent pandemic robot swabbing technology was not available yet. With our work, we show that in future incidents this technology can be a helpful tool to maximize testing capacity and human safety at the same time. While the results of this first clinical study of 52 test subjects are statistically significant, SR-NOCS can also be applied during seasonal influenza, screening for multiresistant germs before hospital admission or throat infections. Currently, two SR-NOCS stations can be operated by a single HCW or a trained person. One short-term goal is to increase this ratio to five SR-NOCS operated by one HCW. Furthermore, SR-NOCS can be further optimized by combining complementary laboratory equipment and further automating testing and screening procedures. Finally, deploying a statistically significant number of screening stations that generate enough diagnostic data would presumably allow data-based Artificial Intelligence (AI) algorithms to efficiently support public bodies in predicting the virus transmission rates and the distributions more accurately. Autonomous systems and robots are of high value not only for screening purposes but also because they can support HCWs and limit exposure to infected patients in manifold situations.

## Conclusion

The presented clinical study showed that force-sensitive and compliant robots may support the intervention and care of patients who rely on physical interaction. Initial results indicate that SR-NOCS is as effective as an HCW swab. The results show a complete positive correlation between autonomous and manual sampling, with a very high test subject acceptance. Beyond that, SR-NOCS reduces the risk of infections and consumes less PPE by inherent design. SR-NOCS is designed to be set up and taken into operation within a few hours. The two experimental studies show that complete samples can be taken within 2 min. Considering the overall test workflow under 24-h operation, one SR-NOCS station (containing one robot) can take more than 300 tests per day in a consistent and repeatable way. This quantity can be readily increased twofold or even threefold at a single station by incorporating multiple swab robots.

## Methods

### Subjects

Thirty-one right-subjects (19 female, 37 ± 11 years of age) took part in Experiment 1, and twenty-one subjects (8 female, 32 ± 16 years of age) took part in Experiment 2 (see Table 1). The study was approved by the Ethics committee of the faculty of medicine of the Technical University of Munich (ID 152/20 S-KH, DRKS00021420, 16.04.2020) and also registered at the World Health Organization. Informed consent from all subjects and/or their legal guardian(s) has been obtained for both study participation and publication of identifying information/images in an online open-access publication. Both experiments were performed under the principles of the Declaration of Helsinki and we confirm that all experiments were performed following relevant guidelines and regulations.

### Procedure

#### Test workflow

The objective of this study was to validate the automation of the swab procedure only. A typical NP swab test includes several tasks besides the swab itself (e.g. opening the test sample, and placing the swab into the sterile tube), which were not automated for this study and could be further automated. Currently, the station is supervised by two to three hospital employees - one to two for interacting with the test subjects and commanding the robot (also called “operator”), and another one for hygiene and handling of test probes. Test subjects were offered to participate in the study before performing the scheduled screening test. The informed consent (detailed above) from all subjects was obtained after a verbal explanation of the study details. Having completed the planned screening test and having received instruction on the test procedure, each subject took a seat and adjusted the chair’s height. Each swab was performed after placing the nostril and mouth on the corresponding fixture. The start of each swab had to be actively confirmed both by the personnel and the test subject for safety reasons and by pressing a pedal on the ground. The operator would observe the procedure and could interrupt it at any time. Each swab could be repeated in case of incorrect procedure (e.g. if the subject moved away from the fixture). A collective swab was sampled from the naso- and oropharynx before proceeding with the probe according to the aforementioned measures. Finally, a questionnaire is filled out by each subject. In the supplementary video S1, an example of a subject trial can be observed, and a complete trial timeline is detailed in the supplementary document S1.

#### NP and OP swab

All healthcare workers collecting NP and OP swab specimens from suspected or confirmed COVID-19 patients involved in this study were well-trained on the procedure and did wear a clean, non-sterile, long-sleeve gown, a medical mask, eye protection (i.e. face shield), and two sets of sterilizable gloves. All procedures were conducted at the screening facilities of Klinikum rechts der Isar with the subject in an upright posture. For initial diagnostic testing, all subjects were sampled with an upper respiratory specimen and swabbed with a synthetic plastic rod at the naso- and oropharynx. The swab was initially inserted into the nose and subsequently advanced towards the posterior wall of the nasopharynx, where the first specimen was collected through rotary manipulations. Following this, the swab was further inserted into the oropharynx, employing the same method to collect the second specimen. Swabs were immediately inserted into sterile tubes containing 2–3 ml of viral transport media and stored upright before being collected and transferred to the Institute of Virology for assessment. For the trial and general screening, MWE E-Transwab (MWE, Corsham Wiltshire, UK) swabs were used. Upon completing the swab collection from an individual, the healthcare workers (HCWs) proceeded to disinfect the outer layer of their gloves using an appropriate disinfectant. Additionally, the HCWs followed a protocol of changing gloves after every fifth subject to maintain hygiene and minimize the risk of cross-contamination. Finally, the same swab was used for both NP and OP procedures as there is no discernible distinction between these two swabs; the choice primarily revolves around comfort. Typically, NP swabs exhibit greater flexibility, whereas OP swabs tend to be stiffer. However, these variances hold little significance regarding the accuracy and effectiveness of testing. The target testing zones are essentially identical, differing only in the approach employed to retrieve a substantial amount of viral RNA, as highlighted in studies by^53,54^.

#### Automated NP and OP swabs

As explained, a significant challenge of such an application is the design of the compliant physical interaction between the robot and the patient. To ensure correct and harmless test performance, the force-sensitive robot is controlled by a Cartesian impedance control algorithm (Eq. 4) that allows the otherwise rigid mechanics to behave as a 6-dimensional translation/rotation spring-damper system between its end-effector pose and its desired pose^52^. Each swab procedure (nose, throat) is split into an approach, a sweep, and a retreat phase. The programmed robot trajectory and the time duration of each phase mimic an ideal swab procedure performed by medical personnel for larger-than-normal anatomic dimensions. The well-designed compliant behavior of the robot guarantees low contact forces between the swab and the patient regardless of anatomic shape. The contact force is accurately derived from the robot joint torque sensors in each joint by a model-base observer and is affected by noise and a configuration-dependent offset value^52^ (see next subsection for more details). Both NP and OP swabbing procedures employ the same impedance controller. These steps were performed consecutively, with subjects positioning their nostril and mouth on the respective fixtures (as depicted in our supplementary video S1). The NP swab was conducted initially, followed by the OP procedures. The entire process was automated sequentially, employing the concept of decision trees. An example of this estimated contact force between the robot and a patient along one test can be seen in Fig. 4. In this case, each swab was performed correctly and no repetitions were necessary. The variability in test length can be seen in the inactive periods (b) until each swab start is confirmed by both the test subject and the operator. The offset and noise can be observed during these idle periods (b) where the robot is in a controlled position and not yet interacting with the human. To obtain a more precise estimate of the contact force, its offset is estimated for each test as the average contact force vector during the static period before the throat swab in the (b) region. The maximum values of the resulting relative contact forces for all tests are listed in Table 3 (in the current application, they take place during the throat swab procedure). In future iterations, variations in interaction positions and forces might be monitored to rate the quality of each test and detect incorrect procedures. In the subsequent subsection, we elaborate on the controller and the procedure for motion generation employed by the robot.

### Robot impedance controller and motion generator

The dynamics of a rigid body manipulator with $n = 7$ degrees of freedom (DoF) are described by the following equation,

1 $${\varvec{M}}({\varvec{q}}){\ddot{\varvec{q}}} + {\varvec{C}}({\varvec{q}},{\dot{\varvec{q}}}) {\dot{\varvec{q}}} + {\varvec{g}}({\varvec{q}}) = {\varvec{\tau }}_{\text{d}} + {\varvec{\tau }}_{\text{ext}}.$$

where, ${\varvec{\tau }}_{\text{d}} \in {\mathbb {R}}^n$ represents the desired actuator torques, $\varvec{q} \in {\mathbb {R}}^n$ represents joint space coordinates, $\varvec{M}(\varvec{q}) \in {\mathbb {R}}^{n\times n}$ is the positive definite inertia matrix, $\varvec{C}(\varvec{q},\dot{\varvec{q}}) \in {\mathbb {R}}^n$ represents the Coriolis and centrifugal components, and $\varvec{g}(\varvec{q}) \in {\mathbb {R}}^n$ are the generalized gravity forces. Finally, ${\varvec{\tau }}_{\text{ext}} \in {\mathbb {R}}^n$ depicts the external torques from the environment contact. The above dynamics can be represented in the task space as follows

2 $${\varvec{M}}_C({\varvec{q}}){{\ddot{\varvec{x}}}} + {\varvec{C}}_C({\varvec{q}},{\dot{\varvec{q}}}) {{\dot{\varvec{x}}}}+{\varvec{g}}_C({\varvec{q}}) = {\varvec{f}}_{\text{d}}+{\varvec{f}}_{\text{ext}},$$

where the subscript ${-}_C$ describe the associated matrices in task space and the respective wrenches in the Cartesian are related through Jacobian matrix $\varvec{J}$ to the joint space torques, i.e.,

3 $$\begin{aligned} {{\mathcal {\varvec{\tau }}}}_{\text{ext}}&= {\varvec{J}}({\varvec{q}})^{T}{\varvec{f}}_{\text{ext}},\\ {{\mathcal {\varvec{\tau }}}}_{\text{d}}&= {\varvec{J}}({\varvec{q}})^{T}{\varvec{f}}_{\text{d}}. \end{aligned}$$

Assuming joint-level gravity compensation, the Cartesian impedance controller is formulated as

4 $$\begin{aligned} {\varvec{f}}\mathrm {_d} =\varvec{M}_C({\varvec{q}}){{\ddot{\varvec{x}}}\mathrm {_d}} + {\varvec{C}}_C({\varvec{q}},{\dot{\varvec{q}}}) {{\dot{\varvec{x}}}\mathrm {_d}}+ \varvec{D}_C(\dot{\varvec{x}}\mathrm {_d} - \dot{\varvec{x}})+\varvec{K}_C(\varvec{x}\mathrm {_d} - \varvec{x}), \end{aligned}$$

where, $\varvec{D}_C$ and $\varvec{K}_C$ represent the damping and stiffness matrices in the task space, respectively^55,52^. The system’s compliance is tuned through these matrices to have desirable interaction at the tip of swab. The symbols $\varvec{x}$ and ${\varvec{x}}_\text{d}$ denote the actual and desired Cartesian positions, respectively. The desired trajectory during the swabbing procedures is a sinusoidal policy. In detail, the robot triggers a sinusoidal motion when $\varvec{f}_{\text {ext}}$ increase up to a predefined threshold $\bar{\varvec{f}}$ ($\varvec{f}_{\text {ext}} > \bar{\varvec{f}}$). Upon detecting this condition, the robot initiates a sinusoidal motion defined by

5 $$\begin{aligned} \varvec{x}_d(t) = \varvec{x}_0 + \varvec{{\bar{x}}} \sin (\omega t + \phi ), \end{aligned}$$

where $\varvec{x}_0$ is its initial position, $\varvec{{\bar{x}}}$ is the amplitude of the sinusoidal motion, $\omega$ is the angular frequency, and $\phi$ is the phase offset. Different values are selected for NP and OP procedures. This trajectory is then followed via (3) and (4).

#### COVID-19 PCR test

Routine confirmation of SARS-CoV-2 infection was based on the detection of unique sequences of virus RNA by real-time reverse-transcription polymerase chain reaction (rRT-PCR) and was conducted by the Institute for Virology and Bacteriology at the Klinikum rechts der Isar, Technical University of Munich. For testing for a COVID-19 infection, nucleic acids (DNA and RNA separated) were extracted on m2000sp Liquid Handler. The Taqman real-time PCR running on a Thermo Fisher 7500 Cyclers was checked with N1 primers of the 2019 Novel-Coronavirus (2019nCoV) rRT PCR Panel and Primer as provided by CDC (CDC, Atlanta, GA 30333). If positive a confirmation test was run against N3 primers of the same panel.

#### Safety and hygienic measures

The two identified main risks of this kind of study are the physical interaction with the robot and the risk of cross-infection. Therefore, several precautionary measures were implemented for this prototypic setup. A plexiglass wall was placed to restrict the robot’s motions mechanically. Direct physical interaction with the test subjects was only possible via a flexible swab through the fixture hole in a restricted motion range. The test subjects controlled the robot’s start (with confirmation from the operator) and could always freely move away from the plexiglass wall. The interaction with the trained hospital employees only occurred if the robot was stopped. To prevent cross-infection, the employees changed gloves, and mouth-and-nose disposable fixtures and fully disinfected the station, robot, and chair surfaces using Perform $\copyright$ fabrics (Schülke & Mayr GmbH, Norderstedt, Germany) between tests. Additionally, the robot’s cover and finger gloves were changed every few tests or if a test subject coughed. The tests were performed in a well-ventilated open garage with the employees and test subjects maintaining a safe distance between themselves.

### Statistics

For case number planning, we assumed a true match probability of 90 %. Based on this assumption and with a case number of 30 subjects, we could estimate an exact 95 % confidence interval (Clopper-Pearson interval) for the match probability. With this presumption, the confidence interval width (“upper limit - lower limit”) does not exceed 25 percentage points. A composite measure of positivity was used as the gold standard; cases included any positive result by rRT-PCR from either specimen type. Viral detection levels between HCW- and SR-NOCS-collected samples were compared using the 2-sided Pearson test for correlation. Descriptive data were outlined in absolute numbers, whereas averages were outlined as medians and mean. Analyses were conducted using IBM SPSS Version 23 (IBM Deutschland GmbH, 71137 Ehningen, Germany).

### Supplementary Information

Supplementary Video 1.

Supplementary Information 2.

## Data Availability

Supplementary information is available for this paper. Correspondence and requests for materials should be addressed to Sami Haddadin (haddadin@tum.de).
