# Supplementary material for: Autonomous swab robot for naso- and oropharyngeal COVID-19 screening
Source: Sci Rep. 2024 Jan 2;14:142. doi: 10.1038/s41598-023-50291-1 (PMC10762007; doi:10.1038/s41598-023-50291-1)
Supplement: Supplementary file 2 — Supplementary Information 2. [file 41598_2023_50291_MOESM2_ESM.pdf]

Supplementary material: Autonomous Swab Robot for Naso- and Oropharyngeal COVID-19 Screening

Simon Haddadin, Dirk Wilhelm, Daniel Wahrmann, Fabio Tenebruso, Hamid Sadeghian, Abdeldjallil Naceri, and Sami Haddadin,

S1 Image and video data

Informed consent from all subjects and/or their legal guardian(s) has been obtained for both study participation and publication of identifying information/images/video in an online open-access publication.

S2 Supplementary video

Video entitled '*Autonomous Swab Robot for Naso- and Oropharyngeal Coronavirus Screening (SR-NOCS)*' The video captures the initial test trials during the pandemic and the first lockdown in Europe (April 2020). It also depicts a full trial run for a patient at the automated COVID-19 test station, as conducted in both experiments.

S3 COVID-19 PCR test

Routine confirmation of SARS-CoV-2 infection was based on the detection of unique sequences of virus RNA by real-time reverse-transcription polymerase chain reaction (rRT-PCR) and was conducted by the Institute for Virology and Bacteriology at the Klinikum rechts der Isar, Technical University of Munich. For testing for a COVID-19 infection, nucleic acids (DNA and RNA separated) were extracted on m2000sp Liquid Handler. The Taqman real-time PCR running on a Thermo Fisher 7500 Cyclers was checked with N1 primers of the 2019 Novel-Coronavirus (2019nCoV) rRT PCR Panel and Primer as provided by CDC (CDC, Atlanta, GA 30333). If positive a confirmation test was run against N3 primers of the same panel.

S4 Safety & hygienic measures

The two identified main risks of this kind of study are the physical interaction with the robot and the risk of cross-infection. Therefore, several precautionary measures were implemented for this prototypic setup. A plexiglass wall was placed to restrict the robot's motions mechanically. Direct physical interaction with the test subjects was only possible via a flexible swab through the fixture hole in a restricted motion range. The test subjects controlled the robot's start (with confirmation from the operator) and could always freely move away from the plexiglass wall. The interaction with the trained hospital employees only occurred if the robot was stopped. To prevent cross-infection, the employees changed gloves, and mouth-and-nose disposable fixtures and fully disinfected the station, robot, and chair surfaces using Perform© fabrics (Schülke & Mayr GmbH, Norderstedt, Germany) between tests. Additionally, the robot's cover and finger gloves were changed every few tests or if a test subject coughed. The tests were performed in a well-ventilated open garage with the employees and test subjects maintaining a safe distance between themselves.

S5 Data availability statement

The study has been registered to the German Registry of Clinical Studies (DRKS) with the ID DRKS00021420 (www.drks.de). The study is also approved by the WHO Registry (http://apps.who.int/trialsearch/). The datasets generated and/or analyzed during the current study will be available via these repositories.

S6 Summary of the State of the Arts contributions

Table S1 summarizes key attributes from state-of-the-art papers referenced and discussed in our manuscript. Each paper is assigned a unique reference number, and the 'System Characteristics' column provides a concise overview of the essential features, design, and control mechanisms of the discussed robotic systems. Categorization in the 'Type of Study' column offers insights into methodologies, encompassing various study types. 'Clinical Study' indicates direct engagement with patients or volunteers. 'In Hospital' and 'In Public' describe testing environments, while 'Autonomous' and 'Teleop' signify system automation levels. 'Number of Participants' quantifies the study scale, 'Force Measurement' highlights sensitivity, and 'TRL' (Technology Readiness Level: further information can be found on this link: https://www.twi-global.com/locations/deutschland/was-wir-tun/haeufig-gestellte-fragen/was-sind-technology-readiness-levels-trls) reflects the developmental maturity of the systems. This detailed table enhances accessibility, providing a valuable resource for readers to navigate and understand the nuances of each study. Further abbreviation on the table: 'NF' refers to Data Not Found in the cited paper.

S7 Trial Timeline

Figure S1 illustrates the flow of the automated tests being studied (current iterations, evaluated in this study). In the clinical study, the initial step (registration) is manually performed by the scientific staff due to the limited number of subjects (a detailed description of the subject information sheet is provided). Before a subject enters the test station, the station is prepared and cleaned by the scientific staff. This process includes removing the nose and mouth adapter, immersing it in disinfectant liquid following the manufacturer's instructions, and conducting a thorough surface disinfection of the Plexiglas disc, chair, robot, and gripper. The sterile nose and mouth adapter is then reinserted before releasing it for the next test. The test station comprises two adjacent cubicles physically separated by a wall. Typically, two subjects are accommodated per test run, and their scheduling is coordinated to avoid overcrowding in the waiting area. Subjects are called into the study room one at a time, with subject 2 entering once subject 1 is seated. After the test run, subjects exit the study room sequentially to prevent direct contact between patients at a microscopic level. Macrologistics and waiting room administration are not within the scope of this study. However, we will analyze the impact of parallelization and micrologistic measures on waiting times to optimize patient organization during clinical deployments. The main steps are outlined and demonstrated in Figure S1. These steps correspond to those performed at the existing prototypical station. Here below we detail those nine steps depicted in Figure S1.

S7.1 Registration and test kit handover

Easy-to-understand instructions about the study procedure, including manual registration and contactless handover of the test kit.

S7.2 Open test kit

Before opening the test kit, the individual must wash and disinfect their hands. The process of opening the kit is demonstrated in advance, and each component is explained. The kit includes disposable gloves, a mouthpiece, a nosepiece, a mask, and disinfection materials.

Table S1: Table summarizing the current advancements in swabbing systems for comparative analysis with our work.

Paper	System Characteristics	Study Type	Clinical Study (in hospital)	In public	Autonomous	Teleoperation	Nr. of subjects	Force measurement	TRL
-41	hybrid rigid-soft flexible robot for NP and OP, 3-D fiber Bragg grating, constrained compliance control	System implementation with demonstrated proof of concept	No	No	No	Yes	0	Yes	3
-42	Robotic remote manipulation platform for NP and OP, force control, visual confirmation	System implementation with experimental study in laboratory	No	No	No	Yes	16	Yes	4
-43	Teleoperated OP system, remotely operated, hybrid force/position control	System implementation and clinical study	Yes	No	No	Yes	20	Yes	6
-44	Teleoperated NP sampling robot with variable remote center of motion (RCM) constraint	System implementation with demonstrated proof of concept	No	No	No	Yes	0	Yes	3
-45	visual servoing control framework for automated NP, deep learning-based nostril detection, axial control, and System implementation with demonstrated proof of concept	No	No	Yes	No	0	No	3	
-46	Back drivable spherical wrist with for rendering human wrist-like capabilities, hybrid motion-impedance control, Application in OP	System implementation & laboratory experiment	No	No	Yes	No	0	Yes	2
-47	Design and analysis of a pneumatic-actuated soft NP swab (V-tube)	System implementation & laboratory experiment	No	No	-	-	0	No	2
-48	9 DOF rigid-flexible coupling robot for OP, visual system, force-sensing,	System implementation with demonstrated proof of concept, validated on volunteers	No	No	Yes	No	NF	Yes	4
-49	Autonomous robot for OP, multiple manipulators, machine vision, computer vision based	System implementation with demonstrated proof of concept, validated on volunteers	No	No	Yes	No	NF	Yes	2
-50	Autonomous OP robot, throat detection algorithm, force/position hybrid control	System implementation and experimental study & clinical study	Yes	No	Yes	No	14	Yes	6
-51	Autonomous NP robot, force restriction mechanism, RCM mechanism	System implementation with demonstrated proof of concept	No	No	Yes	No	0	Yes	3
Ours	Supervised autonomous NP and OP swabbing station, collaborative tactile robot, impedance controller	System design & Implementation, prototype & clinical study	Yes	Yes	Yes	No	52	Yes	7

S7.3 Post-disinfection by test subjects

After the technical staff demonstrates the disinfection process, the test subject is responsible for disinfecting their station. They should use the disinfection materials provided in the test kit, along with the gloves and mask.

S7.4 Inserting the nose disposable fixation

The test subject is trained to insert the disposable nose fixation while wearing gloves.

S7.5 Inserting the mouth/throat disposable fixation

the test subject is trained to insert the disposable mouth/throat fixation while wearing gloves.

S7.6 Nasal swab left

The test is initiated following instructions from qualified personnel. Each examination step and robot movement can only be initiated by pressing the foot pedal. Once the robot reaches the test starting position, the subject should place their nose on the nose cap and begin the test by pressing the foot

pedal. The subject can interrupt the test at any time by pressing an emergency stop button to halt the robot. Additionally, the test subject has the option to make unlimited evasive movements to the rear at any time.

S7.7 Nasal swab right

Similar procedure as point S7.6.

S7.8 Throat swab

The test is initiated following instructions from qualified personnel. Each examination step and robot movement can only be initiated by pressing the foot pedal. Once the robot reaches the test starting position, the subject should place their throat on the mouth/throat fixation and begin the test by pressing the foot pedal. The subject can interrupt the test at any time by pressing an emergency stop button to halt the robot. Additionally, the test subject has the option to make unlimited evasive movements to the rear at any time.

S7.9 Cleaning and fixations removal by test subjects

After a successful test procedure, the test subject should remove the disposable mouth and nose fixations, pre-disinfect their station, and dispose of all used materials. Subsequently, qualified personnel will clean and disinfect the test station. The station operator ensures the complete removal of the nose and mouth fixations, immerses them in disinfectant liquid following the manufacturer's instructions, and conducts a thorough surface disinfection of both sides of the Plexiglas disc, the chair, the robot, and the gripper. Swabs are collected and sent to the laboratory. Finally, the sterile nose and mouth adapter is replaced and prepared for the next test.

S8 Data Availability

Correspondence and requests for materials should be addressed to Sami Haddadin (haddadin@tum.de).

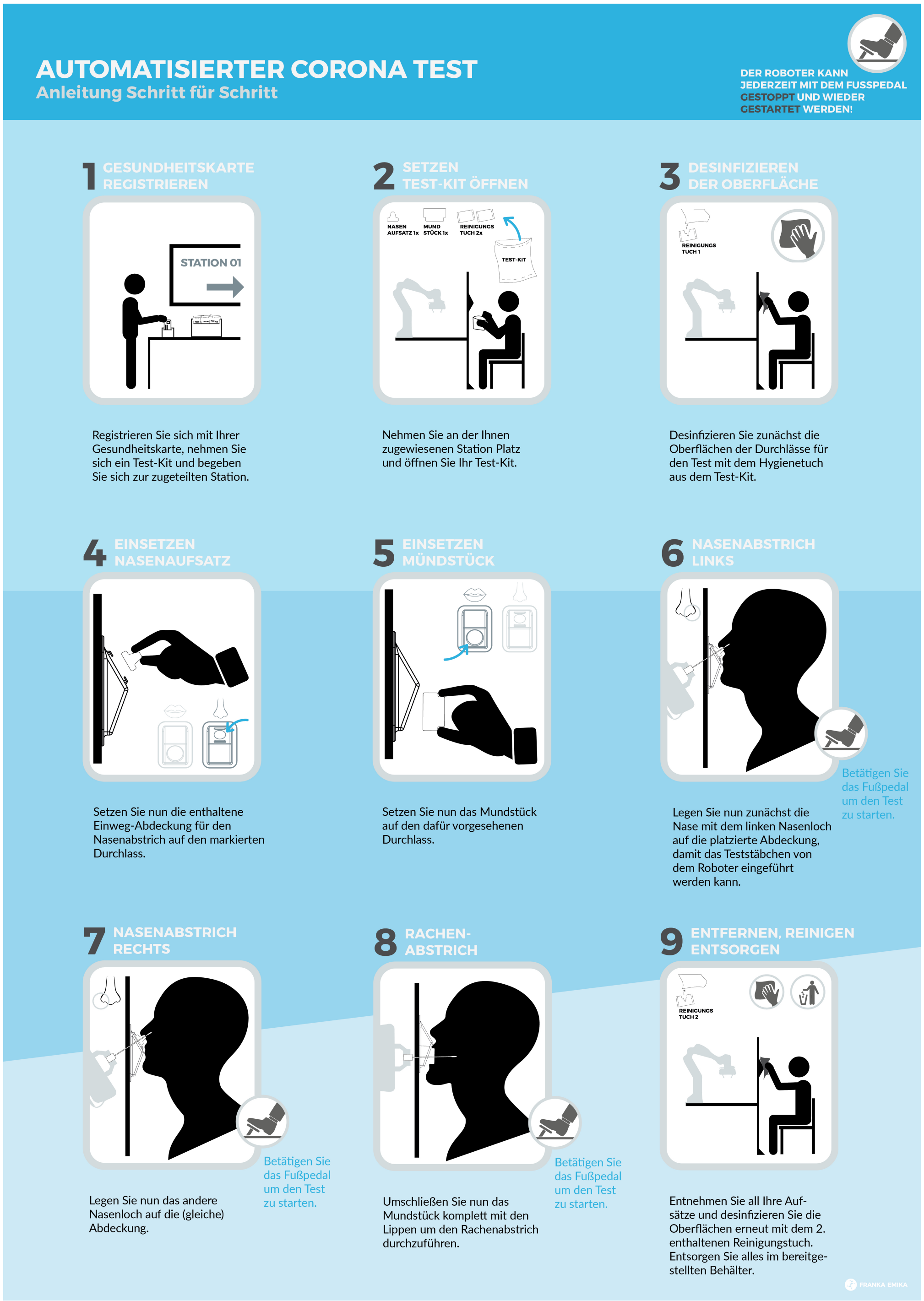

Figure S1: Steps within single trial timeline.
